# Supplementary material for: Parcels and particles: Markov blankets in the brain
Source: Netw Neurosci. 2021 Mar 1;5(1):211–51. doi: 10.1162/netn_a_00175 (PMC7935044; doi:10.1162/netn_a_00175)
Supplement: Supplementary file 1 [file netn-05-211-s001.pdf]

Friston, K. J., Fagerholm, E. D., Zarghami, T. S., Parr, T., Hipólito, I., Magrou, L. & Razi, A. (2021). Supporting information for "Parcels and particles: Markov blankets in the brain." *Network Neuroscience*, 5(1), 211–251. https://doi.org/10.1162/netn_a_00175

Supplementary Material

Parcels and particles: Markov blankets in the brain

*Karl J. Friston[1], Erik D. Fagerholm[2], Tahereh S. Zarghami[3], Thomas Parr[1], Inês Hipólito[4], Loïc Magrou[5] and Adeel Razi[1,6]**

[1]*The Wellcome Centre for Human Neuroimaging, University College London, Queen Square, London WC1N 3AR. UK*

[2]*Department of Neuroimaging, King's College London, SE5 8AF, UK*

[3]*Bio-Electric Department, School of Electrical and Computer Engineering, University of Tehran, Amirabad, Tehran, Iran*

[4] *Berlin School of Mind and Brain & Institut für Philosophie Humboldt-Universität zu Berlin, Germany.*

[5] *Univ Lyon, Université Claude Bernard Lyon 1, Inserm, Stem Cell and Brain Research Institute U1208, 69500 Bron, France*

[6] *Turner Institute for Brain and Mental Health, Monash University, Clayton, Australia*

* Correspondence to: Adeel Razi (adeel.razi@monash.edu)

E-mails: Karl Friston k.friston@ucl.ac.uk; Erik Fagerholm erik.fagerholm@kcl.ac.uk; Tahereh Zarghami tzarghami@ut.ac.ir; Thomas Parr thomas.parr.12@ucl.ac.uk; Ines Hipolito inesh@uow.edu.au; Loïc Magrou loic.magrou@gmail.com; Adeel Razi adeel.razi@monash.edu

Markov blankets for random dynamical systems

Formally, the definition of Markov blankets, in terms of dynamical (i.e., causal) influences, is a little more delicate than their definition given a probabilistic graphical model (i.e., conditional dependencies). This is because the conditional dependencies among the states of a dynamical system are those that obtain at nonequilibrium steady state, which depends upon dynamical coupling among states in a nontrivial way. The aim here is to identify sufficient conditions that render subsets of states conditionally independent of each another – so that they can be distinguished in a statistical sense.

Definition (*dissipative partition*): a dissipative partition is a partition into external, blanket (i.e., sensory and active) and internal states, where internal and external states do not influence each other – and one or more subset of states is dissipative, i.e., the leading diagonal elements of the associated Jacobian are large and negative.

Lemma (*Markov blankets)*: The sensory and active states of a *dissipative* partition constitute a Markov blanket $b = (s, a)$ that renders external and internal states conditionally independent:

$$(\mu \perp \eta)|b \Leftrightarrow p(\mu, \eta|b) = p(\mu|b)p(\eta|b) \tag{S1}$$

Proof: at nonequilibrium steady state, the following solution to the Fokker Planck equation holds (Ao, 2004; Qian & Beard, 2005):

$$f(x) = (Q - \Gamma)\nabla\Im(x) \tag{S2}$$

Here, $\Im(x) = -\ln p\,(x)$ is surprisal or self-information and the antisymmetric (skew) matrix $Q = -Q^{\dagger}$ mediates solenoidal flow. The positive definite matrix $\Gamma \propto I$ is a diffusion tensor describing the amplitude of random fluctuations. In this (Helmholtz) decomposition, the flow $f(x)$ can be decomposed into dissipative gradient flows $-\Gamma\nabla\Im$ and divergence free or

solenoidal flow $Q\nabla\Im$. Differentiating, with respect to the states, evinces the relationship between the flow – specified by a Jacobian $J = \nabla f(x)$ – and conditional independencies – specified by a Hessian $H = \nabla^2\Im$:

$$\begin{aligned} \nabla f(x) &= (Q - \Gamma)\nabla^2\Im(x) \Rightarrow \\ J(x) &= (Q - \Gamma)H(x) \Rightarrow \\ H(x) &= -(\Gamma - Q)^{-} J(x) \approx -(\Gamma + Q)J(x) \end{aligned} \tag{S3}$$

Here, the coupling is encoded by the Jacobian (Note: The approximate equality follows from a first-order Taylor expansion of the inverse of a mixture of matrices.). For example, if the Jacobian encoding the coupling between external and internal states is zero, we can express the flow of internal states as a function of, and only of, particular states:

$$J_{\mu\eta} = \nabla_\eta f_\mu(x) = 0 \Rightarrow f_\mu(x) = f_\mu(\pi) \tag{S4}$$

Similarly, the Hessian or curvature matrix encodes conditional dependencies, in the sense that if the corresponding submatrix is zero, internal and external states are conditionally independent:

$$H_{\mu\eta} = \nabla_{\mu\eta}\Im(x) = 0 \Rightarrow \Im(\mu|b,\eta) = \Im(\mu|b) \Rightarrow (\mu \perp \eta)|b \tag{S5}$$

Equation (S3) shows that the amplitude of random fluctuations and solenoidal coupling play a key role in relating dynamic coupling and conditional dependencies. The solenoidal components are especially important in the setting of nonequilibrium steady state. Indeed, on one reading of nonequilibrium dynamics, the very presence of solenoidal flow is sufficient to break detailed balance – and preclude any equilibria in the conventional (statistical mechanics) sense (Ao, 2005; Kwon & Ao, 2011; Seifert, 2012; Zhang et al., 2012).

The symmetry of the Hessian matrix places linear constraints on the solenoidal coupling (Qian & Beard, 2005); where, dropping the dependency on x for simplicity:

$$
\begin{aligned}
&(Q-\Gamma)^{-1}J=\Pi=\Pi^{T}=J^{T}(Q-\Gamma)^{-T}\\
&\qquad\Rightarrow\\
&JQ+QJ^{T}=\Gamma J^{T}-J\Gamma\Rightarrow\\
&\quad vec(Q)=(I\otimes J+J\otimes I)^{-}vec(\Gamma J^{T}-J\Gamma)
\end{aligned}
\tag{S6}
$$

These constraints mean that the solenoidal flow can be expressed as a function of the Jacobian and the amplitude of random fluctuations, as shown in the last equality of equation (S6). In turn, this means we can express the Hessian, encoding conditional independencies, as a function of the Jacobian. For example, in a system with one external, blanket and active state, substituting equation (S6) into equation (S3) gives:

$$
\begin{aligned}
&H(x)\\
&=\begin{bmatrix}
\frac{64\kappa^7+\cdots}{64\kappa^6+\cdots} & \frac{-32\kappa^6(J_{b\eta}+J_{\eta b})+\cdots}{64\kappa^6+\cdots} & \frac{16\kappa^5(J_{b\eta}J_{b\mu}-J_{\eta b}J_{\mu b})+\cdots}{64\kappa^6+\cdots}\\
\frac{-32\kappa^6(J_{b\eta}+J_{\eta b})+\cdots}{64\kappa^6+\cdots} & \frac{64\kappa^7+\cdots}{64\kappa^6+\cdots} & \frac{-32\kappa^6(J_{b\mu}+J_{\mu b})+\cdots}{64\kappa^6+\cdots}\\
\frac{16\kappa^5(J_{b\eta}J_{b\mu}-J_{\eta b}J_{\mu b})+\cdots}{64\kappa^6+\cdots} & \frac{-32\kappa^6(J_{b\mu}+J_{\mu b})+\cdots}{64\kappa^6+\cdots} & \frac{64\kappa^7+\cdots}{64\kappa^6+\cdots}
\end{bmatrix}\\
&J(x)=\begin{bmatrix}
J_{\eta\eta}-\kappa & J_{\eta b} & \\
J_{b\eta} & J_{bb}-\kappa & J_{b\mu}\\
 & J_{\mu b} & J_{\mu\mu}-\kappa
\end{bmatrix},\quad \Gamma=\begin{bmatrix} I & & \\ & I & \\ & & I\end{bmatrix}
\end{aligned}
\tag{S7}
$$

Here, the elements of the Hessian have been expressed as rational functions (ratios of polynomials) of $\kappa > 0$, retaining the leading orders (Note: We have eliminated the amplitude of random fluctuations in the above expressions by assuming, without loss of generality, the states have been suitably scaled to render $\Gamma = I$. Furthermore, to simplify the (symbolic) maths, we have used the

Taylor approximation in equation (S3)). These functions have horizontal and linear asymptotes, such that in the limit of dissipative flows, we have:

$$\lim_{\kappa\to\infty} H(x) = \begin{bmatrix} \kappa & -\tfrac{1}{2}(J_{b\eta}+J_{\eta b}) & 0 \\ -\tfrac{1}{2}(J_{b\eta}+J_{\eta b}) & \kappa & -\tfrac{1}{2}(J_{b\mu}+J_{\mu b}) \\ 0 & -\tfrac{1}{2}(J_{b\mu}+J_{\mu b}) & \kappa \end{bmatrix} \Rightarrow$$

$$\lim_{\kappa\to\infty} H_{\eta\mu}(x) = 0 : \forall x \Rightarrow (\mu \perp \eta) \,|\, b \qquad \text{(S8)}$$

In short, for sufficiently dissipative systems, the linear constraints on solenoidal flow ensure conditional independence between internal and external states, given blanket states.

The above proof assumed single states; however, the results can be generalised using numerical analyses (or symbolic maths) for high dimensional systems. An example is presented in **Error! Reference source not found.**

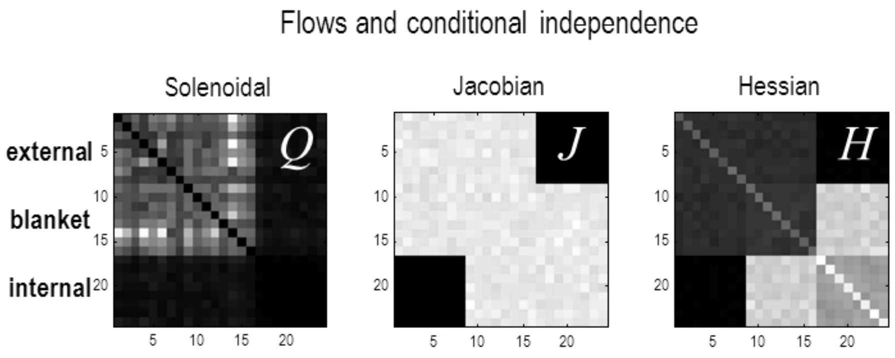

Figure S1

Dissipation and conditional independence: numerical analyses that show the conditional independence between internal and external states depends upon dissipation, as quantified by the average value of the leading diagonal Jacobians. In this example, a system with 24 states was divided equally into external, blanket, and internal states. The panels above report the variance of the solenoidal term, the Jacobian and Hessian, based on 512 random samples where

each element of the Jacobian was sampled from a unit Gaussian distribution and values of 4, 4 and 32 were added to the leading diagonal for the external, blanket and internal states, respectively. The black patches on the lower left (and upper right) shows that an absence of coupling in the Jacobian – between the external and internal states – precludes solenoidal coupling and renders the external and internal states conditionally independent.
